# Statistical Research on the Bioactivity of New Marine Natural Products Discovered during the 28 Years from 1985 to 2012

**DOI:** 10.3390/md13010202

**Published:** 2015-01-07

**Authors:** Yiwen Hu, Jiahui Chen, Guping Hu, Jianchen Yu, Xun Zhu, Yongcheng Lin, Shengping Chen, Jie Yuan

**Affiliations:** 1 Guangdong Province Key Laboratory of Functional Molecules in Oceanic Microorganism (Sun Yat-sen University), Bureau of Education, Guangzhou 510080, China; E-Mails: hyw6@mail2.sysu.edu.cn (Y.H.); huguping@mail.sysu.edu.cn (G.H.); yujchen@mail2.sysu.edu.cn (J.Y.); ceslyc@mail.sysu.edu.cn (Y.L.); 2 Zhongshan School of Medicine, Sun Yat-sen University, Guangzhou 510080, China; 3 Key Laboratory of Tropical Disease Control (Sun Yat-sen University), Ministry of Education, Guangzhou 510080, China; E-Mails: chenjh96@mail.sysu.edu.cn (J.C.); zhuxun8@mail.sysu.edu.cn (X.Z.); 4 School of Chemistry and Chemical Engineering, Sun Yat-sen University, Guangzhou 510275, China; 5 School of Marine Sciences, Sun Yat-sen University, Guangzhou 510006, China

**Keywords:** bioactivity, marine natural products, quantitative analysis, novel compounds

## Abstract

Every year, hundreds of new compounds are discovered from the metabolites of marine organisms. Finding new and useful compounds is one of the crucial drivers for this field of research. Here we describe the statistics of bioactive compounds discovered from marine organisms from 1985 to 2012. This work is based on our database, which contains information on more than 15,000 chemical substances including 4196 bioactive marine natural products. We performed a comprehensive statistical analysis to understand the characteristics of the novel bioactive compounds and detail temporal trends, chemical structures, species distribution, and research progress. We hope this meta-analysis will provide useful information for research into the bioactivity of marine natural products and drug development.

## 1. Introduction

Marine natural products exhibit a wide range of biological activities, which play an important role in the discovery of leads for the development of drugs for the treatment of human diseases [1]. Every year, hundreds of new compounds are found from marine organisms. Since 2008, more than 1000 new compounds are discovered each year. Because of the peculiarity of the marine environment, marine bioactive compounds usually have unique chemical structures and high biological activity. The novel structures and metabolism (biosynthesis) pathways fascinate chemists, and the biological activities of these compounds are interesting to the drug development community [2]. Indeed, there are already some detailed reviews on marine natural products [2,3,4,5,6,7,8,9,10,11] with some focusing on compounds with anticancer [12,13,14,15,16,17] and antibacterial activities, and effects against other infectious diseases [18,19,20,21,22,23,24,25,26,27].

Consequently, the main objective of this article is to cumulatively record the bioactivity of new compounds discovered from marine organisms annually and to reference the 30 review articles published from 1984 to 2014 [28,29,30,31,32,33,34,35,36,37,38,39,40,41,42,43,44,45,46,47,48,49,50,51,52,53,54,55,56,57]. These review articles cover over 17,900 original research articles reporting novel marine compounds. This paper is also an updated and expanded version of the paper that was published in this journal in 2011 [58], which performed a quantitative analysis for these marine natural products. The main difference from the previous article is the focus of this paper.

Here, we detail bioactive compounds discovered from marine organisms from 1985 to 2012. These are analyzed by year, bioactivity, chemical structure, and biological source. All of these aspects of the marine natural products give us a systematic understanding of the marine bioactive compounds.

## 2. Results and Discussion

### 2.1. Temporal Trend of Bioactive Compounds by Year

**Figure 1:**
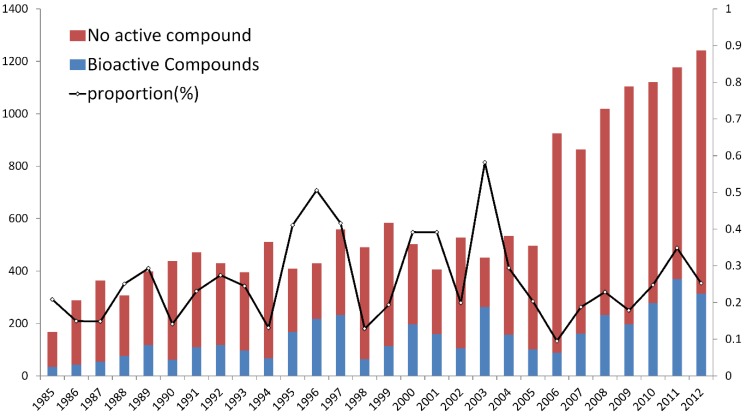
Variation in number of new marine natural products for 1985–2012.

We analyzed temporal trends of the number of annualized novel bioactive products obtained from marine organisms from 1985 to 2012 and compared the total number of the compounds found from marine organisms (Figure 1). We did not include data before 1985, because the novel products discovered annually were less than 100 in those years [58]. As in our earlier analysis, the number of novel marine compounds greatly increased after 1985. Since then, the number stabilized at about 500 products per year in the late 1990s. The second sharp rise in yield was found after 2005 (Figure 1).

In contrast with the two-year cycle fluctuations of discovery of total novel compounds, the bioactive compounds had a volatile period of approximately 3–5 years. For example, after the historical peak of 1989, a second peak occurred in 1992. The third peak was five years later during 1996–1997. Subsequent peaks occurred in 2000 and 2003, but since 2006 the total number of novel compounds discovered annually has seen a huge increase, however the number of bioactive compounds did not increase significantly.

Furthermore, if we compare the proportion of bioactive compounds to total compounds, we see that the annual proportion is not constant. In the proportion curve, the last peak occurred about 10 years ago. The relative analysis was presented by Blunt *et al*. in 2006 [49]. They compared the relative proportion of articles reporting bioactivity (“bioarticles”) between 2003 and 2004 and mentioned that greater attention was being paid to searching for bioactive compounds. However, the data observed in the last decade suggests that the average proportion of bioactive compounds among the new compounds is declining, though there is a large number of marine natural products yet to be explored. This may indicate that the research level of bioactivity is not keeping up with the discovery of new compounds. Thus, the research tools and methods for finding bioactivity need to be improved.

Several advances in technologies can facilitate this. Numerous compounds were reported from easy-to-access, near-shore collected marine samples [51]; advancements in sampling techniques and the work of investigation of bioactivity need to be in close cooperation with compound mining. The first improvement is about methods of bioactivity-guided separation and purification of marine secondary metabolites, which combine the discovery of new compounds with bioactivity assays [59]. These improvements in technology are dependent upon the automation in NMR and mass spectroscopy (MS), which also allow the study of the functions of new compounds extracted from marine organisms’ secondary metabolites [60,61,62]. Second, for the discovery of new lead compounds for drug development, empirical cell-based screening for cytotoxicity has evolved to a more mechanistic approach that targets specific molecular lesions though there are still shortcomings [63]. Combined with phenotypic and high content screening through a large number of drug targets, bioactivity research will be very effective in revealing the potentially useful biological properties of natural products [8]. In addition, a high-throughput strategy was established to sequence and assemble single-cell genomes of novel uncultivated microorganisms from marine samples [64,65]. Using single-cell genomic approaches allows us to study uncultured microbes with partial to near complete genomes. The highly accurate sequence data will generate new knowledge in several areas relevant to medicinal chemistry including the identification and validation of new drug targets in human pathogens. Furthermore, the discovery of new bioactive compounds from marine metabolites will form the basis for new drug leads [66]. As mentioned above, these technological advances are crucial to remove the bottlenecks slowing the discovery of potent secondary metabolites. More interdisciplinary approaches and innovative manipulations are urgently needed to improve bioactivity studies.

In addition, it is worth mentioning that, in this article, the number of bioactive compounds is somewhat imprecise because the bioactivity record updates and the discoveries of new bioactivity on these compounds cannot be reflected in our data. Of the 16617 new compounds, the number of bioactive compounds is 4196, or 25.25%. However, this does not mean that the other 74.75% are inactive. Indeed, their bioactivity may be discovered in future studies.

Some promising lead compounds were found to have no bioactivity in their initial studies. Hymenialdisine, isolated from the marine sponges *Acanthella* sp. and *Axinella* sp. in 1982 [67,68,69,70], did not draw significant attention until it was characterized in 1997 as an nuclear factor kappa-light-chain-enhancer of activated B cells (NF-κB) inhibitor. In 2000 it was shown to be an ATP competitive inhibitor of multiple kinases. Thus, the new compounds will absolutely compose an abundant resource for future bioactivity research and drug development. In recent years, studies of marine natural product have yielded a considerable number of drug candidates [7]. One of these compounds, trabectedin (ET-743 or Yondelis^®^, Madrid, Spain), originally isolated from the Caribbean marine tunicate *Ecteinascidia turbinata*, is a striking example. It has been given marketing authorization from the European Medicines Agency (EMA) and elsewhere for the treatment of advanced soft tissue sarcoma [71,72,73,74,75,76]. Although the percentage of marine natural products that can be developed into drugs is low, it is clear that an increasing number of marine bioactive compounds will be approved for the treatment of human diseases [7,12,77].

**Figure 2:**
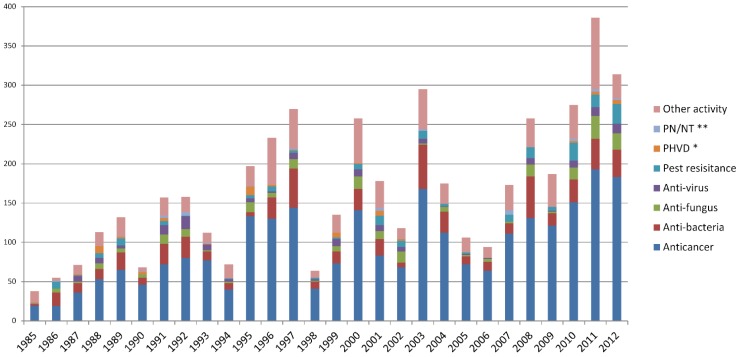
Statistics of bioactivity of new marine natural products by year (* PHVD: Prevention of heart and vascular disease, ** PN/NT: Protection of neurons/neurotoxicity).

Further information about the distribution of bioactivity in the annual bioactive compounds is given by dividing the bioactive compounds into eight groups by year (Figure 2). For the eight groups studied here the proportion of each type of bioactivity always appears stable in the annual data. Some bioactivity groups emerged in the annual data (e.g., PHVD, PN/NT), though their number was always small.

As shown in Figure 1, the number of bioactive compounds dramatically changed every year. For example, more than 250 bioactive compounds were reported in 1997, 2000, 2003, 2008, and 2011, but less than 100 in 1990, 1994, 1998, and 2006. This led us to wonder what causes the large fluctuations in the number of bioactive compounds discovered every year. We hypothesized that it is due to the strong fluctuations in the annual number of anticancer compounds because of the high proportion of the anticancer group (56% average). In other words, the question is whether the total number of bioactive compounds is highly dependent on the quantity of anticancer compounds discovered every year.

**Figure 3:**
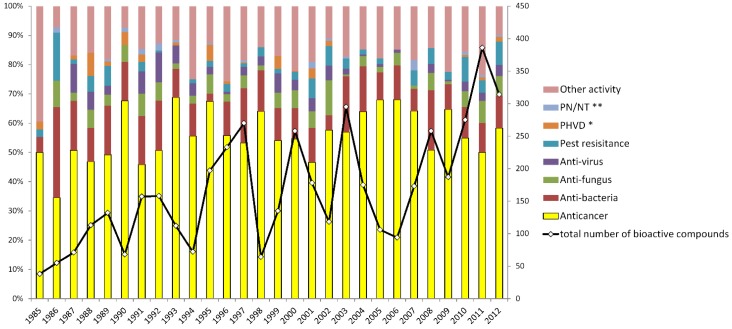
Variation in the number and proportion of the eight bioactivity groups by year (* PHVD: Prevention of heart and vascular disease, ** PN/NT: Protection of neurons/neurotoxicity).

For clarity, we surveyed the percentage of each bioactivity group per year (Figure 3). The total number of bioactive compounds is also shown in Figure 3. Surprisingly, the fluctuations in the proportion of anticancer compounds are not consistent with the annual total number of bioactive compounds. In fact, it is the opposite. In most years, the total number of bioactive compounds negatively correlated with the proportion of anticancer compounds. Especially after 1990 this trend is more pronounced. As shown in Figure 3, in the bioactive compounds-rich years, there were more diverse bioactivity types and a higher number of other bioactive compounds that compressed the proportion of the anticancer group. For example, in 2008 there were more bioactive compounds with a relatively small proportion of antitumor activity. In 2006 or 2009, the situation was just the opposite. This may indicate that if we want to achieve more bioactive marine natural products, we should further develop other types of bioactivity tests beyond anticancer/cytotoxicity assays. However, it is certain that in the past 30 years, anticancer activity such as *in vitro* cytotoxicity tests played the dominant role in bioactivity research of novel marine compounds [49].

### 2.2. The Chemical Structure Distribution of Bioactive Compounds

**Figure 4:**
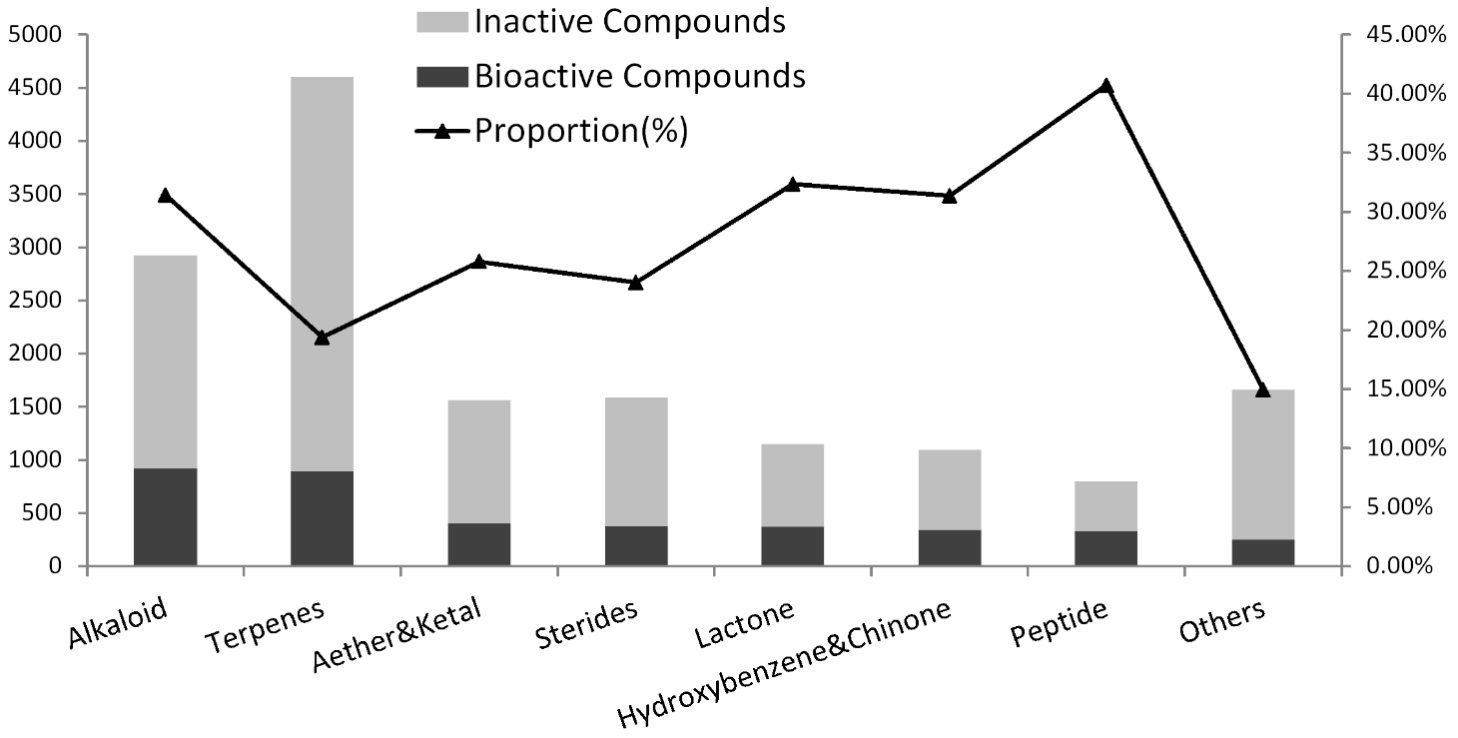
The quantity and proportion of bioactive compounds in each category of chemical compounds.

We divided the compounds into eight categories of chemical structures following the same standard of classification as in the previous article [58]. The number of different chemical compounds varies widely, and the proportion of bioactive compounds in each category is quite different. Figure 4 compares the quantity and proportion of bioactive compounds in each category of chemical compounds. As shown in Figure 4, the highest proportion of bioactive compounds belongs to peptides with 40.85%; this greatly exceeds the average of 28.39%. Natural peptides play central and crucial roles in many physiological/pathological processes. Their reduced size, low immunogenicity, stability, and the powerful strategies for chemical synthesis and/or recombinant expression have made peptides the most promising family of compounds with many potential applications in drug development. Furthermore, their scaffold can be engineered to design compounds with modified biochemical, functional, or biophysical properties [78]. The high activity of peptides provides robust prospects for drug development. An unprecedented number of peptide therapeutics for non-marine sources have been approved [79]. The first drug from the sea, ziconotide (ω-conotoxin MVIIA), is a peptide from a tropical marine cone snail that was approved for chronic pain in the United States in 2004 [80,81]. The success in the discovery and investigation of marine peptides promotes the development of peptide therapeutics.

Lactones are second in proportion with 32.61% followed by alkaloids (31.49%) and hydroxybenzene and quinones (31.36%) in third and fourth place, respectively. Although only 19.39% of terpenes have bioactivity, terpenes are still the second largest bioactive compound class because of the large amount of novel natural products contained in this group.

In many reports, compounds have multiple bioactivities. For example, matemone shows mild cytotoxicity against three cancer cell lines and marginal antibacterial activity against *Staphyloccocus aureus* while also inhibiting division of sea-urchin eggs [82]. Lyngbyabellin B is a novel peptolide isolated from the marine cyanobacterium *Lyngbya majuscula*. Two research teams reported the discovery of lyngbyabellin B concurrently [83,84]. In their reports, lyngbyabellin B is a brine shrimp toxin and antifungal cyclic depsipeptide that exhibits cytotoxicity against KB cells (a human nasopharyngeal carcinoma cell line) and LoVo cells (a human colon adenocarcinoma cell line).

**Figure 5:**
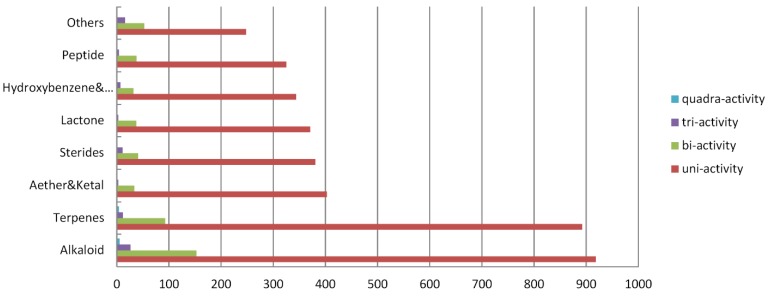
Distribution of multiple bioactive compounds in each category of chemical compounds.

Indeed, marine-derived drugs may have different molecular targets. We analyzed these multi-active compounds in detail. Figure 5 shows the number of uni-activity, bi-activity, tri-activity, and quadra-activity bioactive compounds. The distribution of these compounds is also shown in different chemical classes. To clarify, bi-active compounds were counted twice when the novel compounds were divided into the eight bioactivity groups, and the tri-active compounds were counted three times. When the novel compounds were analyzed by bioactivity or inactivity, the compounds were counted only once.

**Figure 6:**
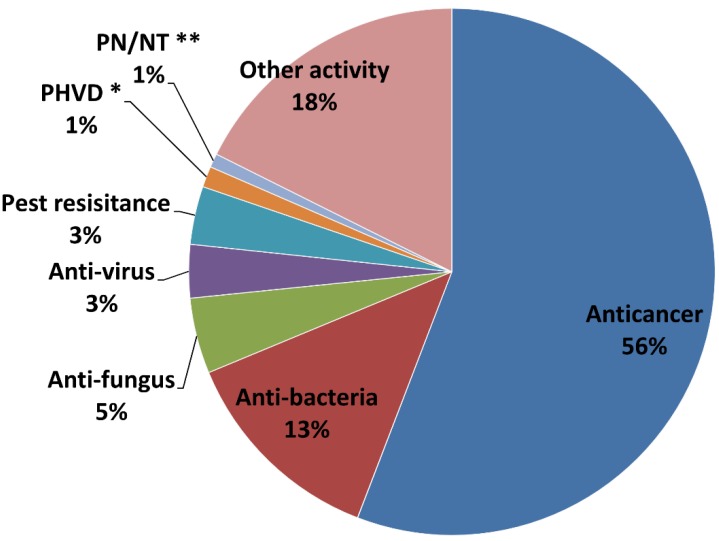
Bioactivities of new marine natural products (* PHVD: Prevention of heart and vascular disease, ** PN/NT: Protection of neurons/neurotoxicity).

### 2.3. Main Bioactivity Groups of the Novel Compounds

Of the 4196 bioactive compounds, 2225 had anticancer activity (56% of the total bioactive compounds; Figure 6). This was followed by antibacterial activity of 521 compounds (13%). The other five groups were represented 14%, including anti-fungus, pest resistance (insect/vermin), anti-virus, PHVD, and PN/NT. The remaining 16% of the bioactive compounds could not be classified into the above bioactive groups and were termed the “other” activity group.

Obviously, anticancer/cytotoxic activity is the dominant group according to Figure 6. However, this does not mean that the anticancer activity is the main bioactivity of marine natural products. The assay outcome of bioactivity of marine natural products is often influenced by multiple factors. First, for long time, huge amounts of scientific research funds spurred into cancer drug discovery and development. Compared to other institutes at the National Institutes of Health (NIH), the US National Cancer Institute (NCI) is in a very favorable funding position. As early as 1974, NCI awarded more money for its research grant programs than all of the other institutes [85]. Beyond the US, during 2006–2007, 153 European public funding organizations spent €603 million on direct cancer drug research and development. Moreover, the 21 US public funding organizations spent €1.7 billion during the same period [86]. Second, big programs can greatly promote the development of marine bioactivity research. In 1983, the NCI established the National Cooperative Drug Discovery Group (NCDDG) program with the aim to discover new synthetic or natural-source derived anticancer drugs [87]. Since then, a large number of marine natural products with antitumor activity have been discovered. Third, the bioactivity assessment is limited by the detection technology. The first largest program on the discovery of bioactive natural products was the Roche Research Institute on Marine Pharmacology in Australia (1974–1981) [88]. A number of promising bioactive marine natural products were reported through the program, but not one anticancer bioactive compound was found, although animal cell lines and human tumor cell lines were used for *in vitro* screening of anticancer drugs as early as the 1950s [89]. This was possible due to of their general pharmacology and gross observations to test marine products’ bioactivity mainly on whole animals apart from specialized functions covering the central nervous, cardio-vascular, and autonomic systems, neurochemical, neurophysiological, and immunological pharmacology [90]. Then in the mid-1980s, NCI implemented a new *in vitro* isease-oriented screen consisting of 60 human carcinoma cell lines representing nine common forms of cancer [91]. Since then, a series of effective colorimetric analyses such as the 3-(4,5-dimethylthiazol-2-yl)-2,5-diphenyltetrazolium bromide (MTT) [92], 2H-Tetrazolium, 2,3-bis(2-methoxy-4-nitro-5-sulfophenyl)-5-[(phenylamino)carbonyl]-hydroxide (XTT) [93], and sulfate reducing bacteria (SRB) [94] assays have provided an efficient detection platform for the anticancer potential of these marine compounds. Testing cell toxicity and anti-proliferative effects has become a universal tool for the detection of bioactive compounds. All of these factors may have had some influence on the anticancer activity as a dominant role in the bioactivity research of novel marine compounds. Furthermore, additional pharmacological testing should be performed to determine whether the cytotoxicity observed with these marine compounds resulted from a specific pharmacologic effect rather than a general toxic effect on the cancer cells used in these investigations [17].

The second largest group encompasses antibacterial compounds. These include squalamine [95], cribrostatins [96], bromosphaerone [97], pestalone [98], and new secondary metabolites that are effective against methicillin-resistant *Staphylococcus aureus* (MRSA) and vancomycin resistant enterococci (VRE) [20]. It is not yet clear if the chemical library of antibacterial compounds will provide a growing number of candidates to select promising leads for extended preclinical assessment. In fact, on the contrary, the challenges to antibacterial discovery have kept the output of novel antibacterial drug classes at extraordinarily low levels over the past 30 years [99,100]. Although an enormous demand exists for new and potent anti-bacterial drugs, because infectious diseases evolve and develop resistance to existing pharmaceuticals [101,102,103], there has also been a marked decline in industrial research aimed at discovering novel antibacterial agents including new drugs that target resistant organisms [104]. The pharmaceutical industry has been withdrawing from research in this area [105].

Although antibacterial tests are relatively inexpensive and easy to perform, natural product screening for novel antibacterials waned due to the low output of good leads. Almost all of the antibiotics approved between the early 1960s and 2000 were synthetic derivatives of the old scaffolds [106]. Other reasons for low output of new and effective antibacterials include: Lack of industry productivity, increasing size of clinical trials, increased generic competition, drug pricing pressure, and marketplace and industry consolidation [104]. After the 1940s–1960s antibacterial discovery has become target oriented and natural product sources has largely been abandoned [100]. Fortunately, significant new advances have been made in the development of target/pathway based on phenotypic screens [107]. Also many innovative techniques such as co-culturing, cross species induction [102] and biofilm development have been adopted [108,109] to confront the challenges of discovery of novel antibacterial agents. This is important to improve the odds of discovery and reduce cost [99]. Beyond technologic approaches, the development of new antibiotics requires innovative funding solutions to promote research in this field [103].

**Figure 7:**
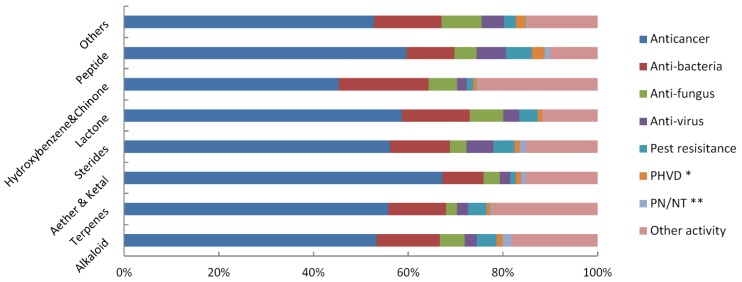
Analysis of the bioactive compounds according to their chemical structures and the proportion of different activities in each type of compound (* PHVD: Prevention of heart and vascular disease, ** PN/NT: Protection of neurons/neurotoxicity).

To measure whether bioactivity is influenced by chemical structure, we analyzed the bioactive compounds according to their chemical structures. As shown in Figure 7, in most chemical groups, the proportion of each bioactivity is equal. For example, the proportion of anticancer activity was close to 50%. Even in the lowest group of hydroxybenzene & quinone, the proportion was 45.31%. Other bioactivities were also present in each group, and their proportions showed no marked changes.

### 2.4. Species Distribution of the Bioactive Compounds

**Figure 8:**
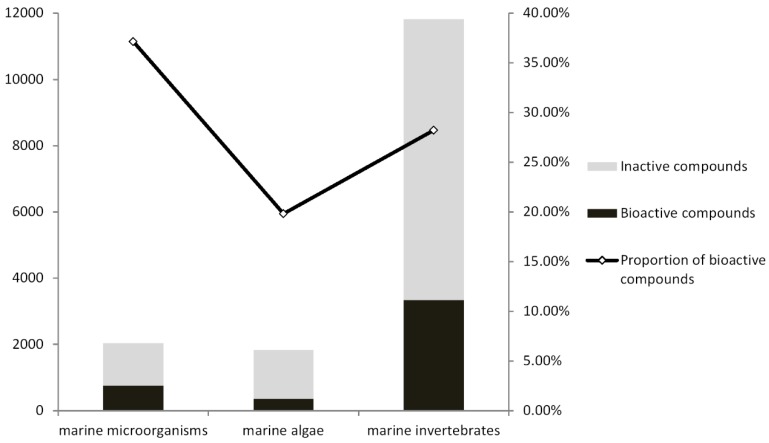
Number of bioactive compounds isolated from the three groups of marine organisms.

**Figure 9:**
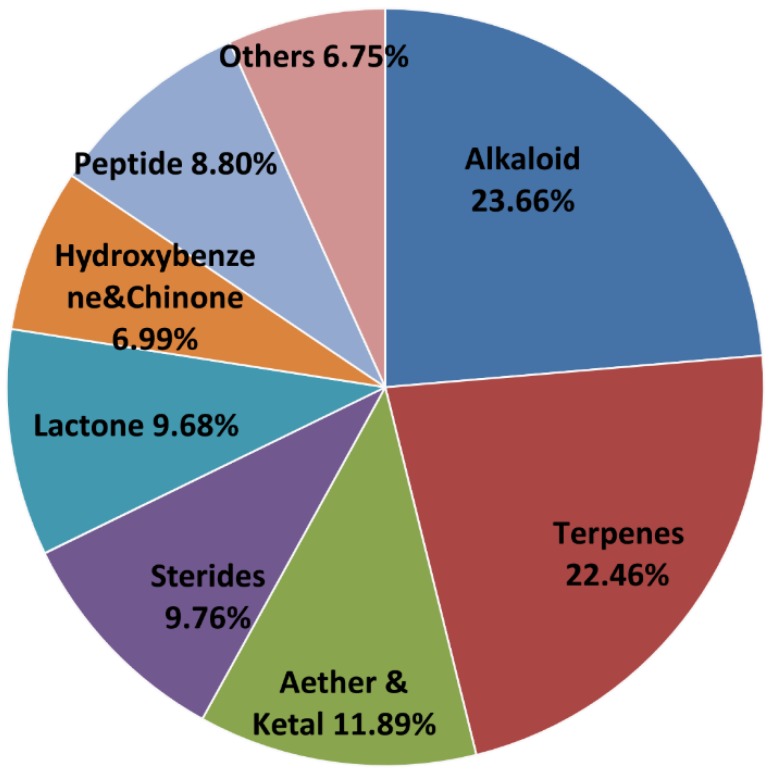
The proportion of bioactive compounds from each group of marine organism with proportions indicated in parentheses.

As reported by Hu [58], approximately 75% of the compounds were isolated from marine invertebrates. Similarly, most novel bioactive compounds were isolated from marine invertebrates (Figure 8), though it is now becoming quite clear that the real producers of many natural products with medical potential are not the organisms from in they were originally discovered [110]. In fact the major bioactive compounds were from Porifera and Cnidaria, accounting for 56.89% of the total bioactive compounds (Figure 9). This is mainly due to the large number of novel compounds found from Porifera (mostly sponge) and Cnidaria (mostly coral), rather than their high proportion of bioactive compounds. Indeed, the proportions of bioactive compounds from Porifera (30.51%) and Cnidaria (23.31%) are just average (28.39%). Porifera and Cnidaria are sessile benthic organisms and easy to collect due to their size, abundance, and color. This explains why Porifera and Cnidaria have attracted the most attention and contribute a large number of metabolites [51]. Sponges pump water to acquire nutrients from the surrounding seawater. They form symbiotic relationships with other species. This metagenome within sponges may be responsible for their numerous biosynthetic pathways and abundant chemical diversity [111]. Furthermore, some symbiotic microbes such as sponge-associated actinomycetes can produce bioactive compounds in parallel. Compounds obtained from marine sponge-associated actinomycetes accounted for 22% of the total natural products from marine actinomycetes [112].

**Figure 10:**
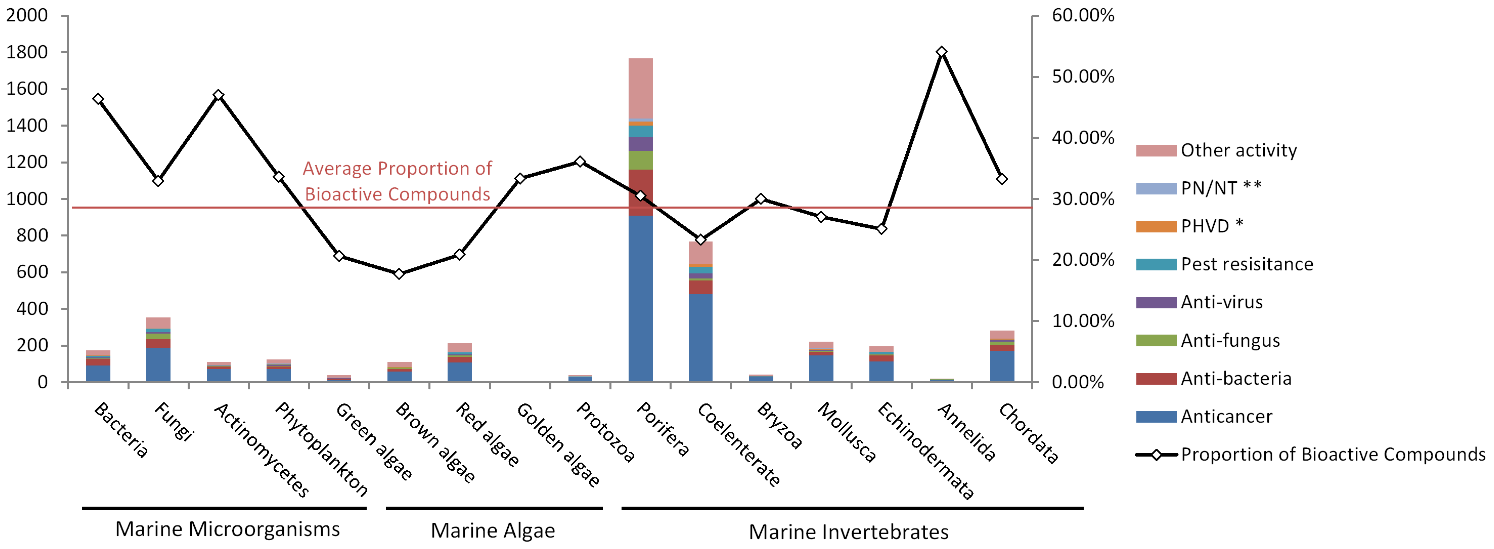
Number and proportion of bioactive/non-bioactive compounds from marine organisms (* PHVD: Prevention of heart and vascular disease, ** PN/NT: Protection of neurons/neurotoxicity).

The types and distribution of bioactivity shown in Figure 10 highlight the distribution of bioactivity in different organisms with no particular preference. This result is in accordance with Figure 2 (by year) and Figure 7 (by chemical structure). The highest proportion of bioactive compounds was discovered from actinomycetes and bacteria. A significant proportion of bioactive compounds from actinomycetes (47.01%) and bacteria (46.38%) is shown in Figure 10. This is much higher than the average proportion (28.39%). This directly contributes to the highest proportion of novel bioactive compounds for marine microorganisms, which is as high as 37.13% versus invertebrates and algae (shown in Figure 8). Marine microorganisms including actinomycetes and bacteria are prolific producers of bioactive substances. There are already some detailed reviews on the capabilities and bioactive potential of marine microorganisms [113,114]. In addition, over 2000 actinomycetes have been isolated from mangrove habitats in China that is a potentially rich source of anti-infectious and anti-tumor compounds as well as agents to treat neurodegenerative diseases and diabetes [115]. The number of bioactive compounds isolated from algae (including green algae, brown algae, red algae, and golden algae) was the smallest. The proportion (19.38%) was also the lowest. Although there is a large algal diversity yet to be explored, the biodiscovery trends observed in the past decade suggest that researchers are focusing their bioprospecting efforts on other targets, such as microorganisms and marine invertebrates [5]. Study of marine natural products from algae requires further emphasis.

## 3. Data and Methods

Data used in this paper are obtained from a marine natural products database established by our research group [116]. This database contains information on more than 15,000 chemical substances, which are mainly abstracted from the annual reviews of Faulkner [28,29,30,31,32,33,34,35,36,37,38,39,40,41,42,43,44,45] and Blunt [46,47,48,49,50,51,52,53,54,55,56,57] about marine natural products. From the original papers, we recorded the biological, chemical, and pharmacological information of every marine natural product. 

In this paper, we use the same classification as the previous article [58]. The bioactive compounds are classified into eight classes according to different types of chemical structure: Terpenoids, steroids (including steroidal saponins), alkaloids, ethers (including ketals), phenols (including quinones), strigolactones, peptides, and others (those that cannot be classified into the above seven classes). We also assign organisms to three major biological classes according to Faulkner and Blunt’s biological classification including marine microorganisms (including phytoplankton), marine algae, and marine invertebrate. Marine microorganisms (including phytoplankton) comprise marine actinomycete, marine fungi, marine bacteria, and phytoplankton. Actinomycete were studied independently because of their unique metabolites. Marine algae include green algae, golden algae, red algae, brown algae, and diatoms. Marine invertebrate include Protozoa, Porifera, Cnidarians, Bryozoa, Molluscs, Echinoderms and Chordates.

According to the data, we divided the compounds into eight different groups of bioactivity. These are anticancer activity, antibacterial activity, prevention of heart and vascular disease (PHVD), protection of neurons/neurotoxicity (PN/NT), anti-fungus activity, pest resistance (insect/vermin), and anti-virus activity. Those bioactive compounds that cannot be classified into the above seven groups or those with less than one specimen per category were classified as “other”. We did not include an anti-inflammatory group, because inflammation underlies many diseases including cancer and neurological damage, such as Alzheimer’s disease [117,118]. Compounds with anti-inflammatory activity were distributed among the above-described eight groups.

## 4. Conclusions

Marine environments present an invaluable source of new natural products that may hold important leads for future drug discovery and development [119]. In this paper, the bioactivities of new marine natural products are analyzed in terms of their temporal trends, chemical structures, species distribution, and research progress. Although the annual number of new natural products has increased year by year, the proportion of new bioactive compounds is falling. As D. John Faulkner said a dozen years ago: “Research on bioactive compounds from marine organisms has provided the bread and butter support of marine natural products research throughout the past quarter century” [10]. Study on the bioactivity of marine natural products should be emphasized to develop new natural compounds from marine organisms.
